# Mandibular incisor inclination and gingival recession after treatment with the Jasper Jumper: a 10-year follow-up

**DOI:** 10.1186/s40510-021-00389-x

**Published:** 2021-12-27

**Authors:** Wilana Moura, José Fernanado C. Henriques, Caroline M. Gambardela-Tkacz, Paula Cotrin, Daniela Garib, Guilherme Janson

**Affiliations:** Department of Orthodontics, Bauru Dental School, University of São Paulo, Alameda Octávio Pinheiro Brisolla 9-75, Bauru, SP 17012-901 Brazil

**Keywords:** Class II malocclusion, Tooth movement, Gingival recession, Orthodontic treatment

## Abstract

**Objective:**

To evaluate the long-term outcomes of Class II treatment with the Jasper Jumper appliance and comprehensive orthodontic treatment concerning inclination of the mandibular incisors and gingival recession.

**Methods:**

Sixteen patients with Class II malocclusion at a mean age of 12.54y (SD = 1.17) were treated with the Jasper Jumper appliance and comprehensive orthodontic treatment. The mean treatment time was 2.05y (SD = 0.21). Dental records were taken before (T1), after treatment (T2) and 11.90y (SD = 0.48) after debonding (T3). The frequency of gingival recession, clinical crown height and mandibular incisor position were evaluated using intraoral photographs, digital models and lateral cephalograms. Interphase changes were evaluated using dependent t and McNemar’s tests. Correlation between clinical crown height and final position of the mandibular incisors was evaluated using Pearson correlation test (*P* < 0.05)**.**

**Results:**

The frequency of gingival recessions increased over time and was observed in 6 (9.4%), 12 (18.8%) and 24 (37.5%) of the mandibular incisors at T1, T2 and T3, respectively. A significant increase in labial inclination and protrusion of the mandibular incisors was observed between T1 and T2 interval. The clinical crown height significantly increased in the follow-up period (T3–T2) and in the complete observation time (T3–T1). There was no correlation between the amount of labial inclination and protrusion of the mandibular incisors and clinical crown height for all time intervals.

**Conclusion:**

No significant correlation between the amount of labial movement of the mandibular incisor and clinical crown height increase was found.

## Introduction

Fixed functional appliances are frequently used for treatment of Class II malocclusion [1–3]. A meta-analysis showed that fixed functional appliances engaged on multibracket systems have mostly dentoalveolar effects rather than skeletal effects for Class II malocclusion correction [4]. When compared to other mechanics such as Class II intermaxillary elastics, fixed functional appliances showed similar effects with predominantly dentoalveolar effects [5–8]. One of the most striking dentoalveolar effects promoted by fixed functional appliances is labial tipping of the mandibular incisors, independent of the growth phase [2, 4].

Gingival recession consists in apical displacement of the gingival margin leading to esthetic problems, dentin hypersensitivity and possible development of erosion and caries lesions [9]. Many factors are associated with gingival recessions and previous studies related to gingival recession and orthodontic movement [10, 11]. A possible explanation for gingival recession after orthodontic treatment is bone dehiscence development related to tooth movement against the labial/buccal bone plate. In this context, labial movement of mandibular incisors promoted by fixed functional appliances would be a predisposing factor for gingival recessions in the long term [10]. According to Garib et al. [12], incisor buccolingual movements are considered the most critical orthodontic movement predisposing to bone dehiscence.

Pancherz and Bjerklin [13] performed a study to analyze the long-term effects of the Herbst appliance on the inclination and alignment of mandibular incisors, evaluating the frequency of gingival recessions. A minimal frequency of gingival recession was found after treatment. Gingival recessions were associated with translation movement of the mandibular incisors and not with the amount of labial tipping. However, there are few studies that evaluated gingival recession after using fixed functional appliances, especially in the long term [2, 3]. Therefore, this study aimed to evaluate long-term outcomes of Class II malocclusion treatment with the Jasper Jumper appliance and comprehensive orthodontic treatment concerning tipping of the mandibular incisors and gingival recessions. The hypothesis was that there is no correlation between mandibular incisor changes after comprehensive orthodontic treatment using the Jasper Jumper appliance, and the increase in gingival recession in the long term.

## Material and methods

The retrospective cohort study was approved by the Ethics Committee in Human Research of the Bauru Dental School, University of São Paulo. (protocol: 2.505.559), and all patients signed an informed consent.

Sample size calculation assumed a correlation of 0.7 [14] between the extent of gingival recession and the inclination of the mandibular incisors to provide a power of 80% with α of 5%. Thirteen participants should be included in the study.

The following inclusion criteria were considered in the pretreatment phase: mixed-race Brazilians, presence of Class II division 1 malocclusion with bilateral minimum severity of one-half Class II molar relationship; presence of convex profile and ANB > 2°; mandibular arch showing minimal or no incisor crowding; early permanent dentition stage; no history of previous orthodontic treatment; and absence of craniofacial anomalies or systemic diseases. The exclusion criteria at posttreatment and follow-up period were: orthodontic treatment not finished with an adequate occlusion, including bilateral molar and canine Class I relationship, presence of crowding or diastemas, inadequate overjet and overbite; absence of a full permanent dentition, except third molars; lack of quality in the record images; dental wear facets; and to follow-up time less than 10 years after debonding.

Initially, 24 subjects attended the inclusion criteria. Seven patients were not found or did not agree to participate. One patient did not have full records with adequate quality. Therefore, the final sample consisted of 16 subjects. The mean age at treatment start (T1) was 12.54y (SD = 1.17; range 10.37–14.57); after debonding (T2), it was 14.59y (SD = 1.17; range 12.73–16.89), and the mean age at the follow-up (T3) was 26.49y (SD = 1.24; range 23.83–28.40). Patients were treated before or during the pubertal growth peak [15], without extractions for a mean period of 2.05y (SD = 0.21; range 1.78–2.86), and the follow-up period was 11.90y (SD = 0.48; range 11.10–12.85) (Table 1). Most patients were skeletal Class II, and four patients were dentoalveolar Class II. The highest value of ANB was 8.2°.

**Table 1:** Characteristics of the sample

Variables	Mean	SD
Initial age (T1)	12.54	1.17
Final age (T2)	14.59	1.17
Follow-up age (T3)	26.49	1.24
Posttreatment time (T2–T1)	2.05	0.21
Follow-up time (T3–T2)	11.90	0.48
Sex	Male	Female
	5 (31.25%)	11 (68.75%)
Skeletal age (T1)	C2	C3
	7 (43.75%)	9 (56.25%)
3X3 Retainer at T3	Presence 13 (81.25%)	Absence 3 (18.75%)

T1—Pretreatment; T2—Posttreatment; T3—Follow-up

C2 and C3—cervical vertebral maturation method

The treatment protocol was performed as described in a previous study [16]. The appliances used in this study were multibracket fixed orthodontic appliances (Roth prescription, Morelli, Sorocaba, SP, Brazil) and the Jasper Jumper appliance (American Orthodontics, Sheboygan, WI, USA). The mandibular arch was tied back to the first or second molars to control the movement of mandibular incisors, resulting in the use of Jasper Jumper appliance. The Jasper Jumper was maintained until overcorrection of Class II anteroposterior discrepancy to a quarter-cusp bilateral Class III relationship. After Jasper Jumper removal, patients were oriented to use Class II intermaxillary elastics ﻿for a mean period of 4 months (ranging from 1 to 8 months) for approximately 14 h. After debonding, retention consisted of a Hawley plate during the day and a Bionator appliance during the night for 1 year. In the mandibular arch, a canine-to-canine fixed retainer was recommended permanently. The mandibular fixed retainer was still present at T3 in 13 out of 16 patients (Table 1).

### Mandibular incisor labiolingual position

Mandibular incisor position was measured on lateral cephalograms using Dolphin software (Dolphin Imaging and Management Systems, Chatsworth, Calif., USA). Magnification of the radiographic images was corrected by the software in 9.8%. The evaluated variables were IMPA (°), L1.NB (°), L1-NB (mm) and L1 to APO (mm) [16]. All variables were measured at the three time points, and interphase changes were calculated (T2–T1, T3–T2 and T3–T1).

### Frequency of gingival recession

**Fig. 1:**
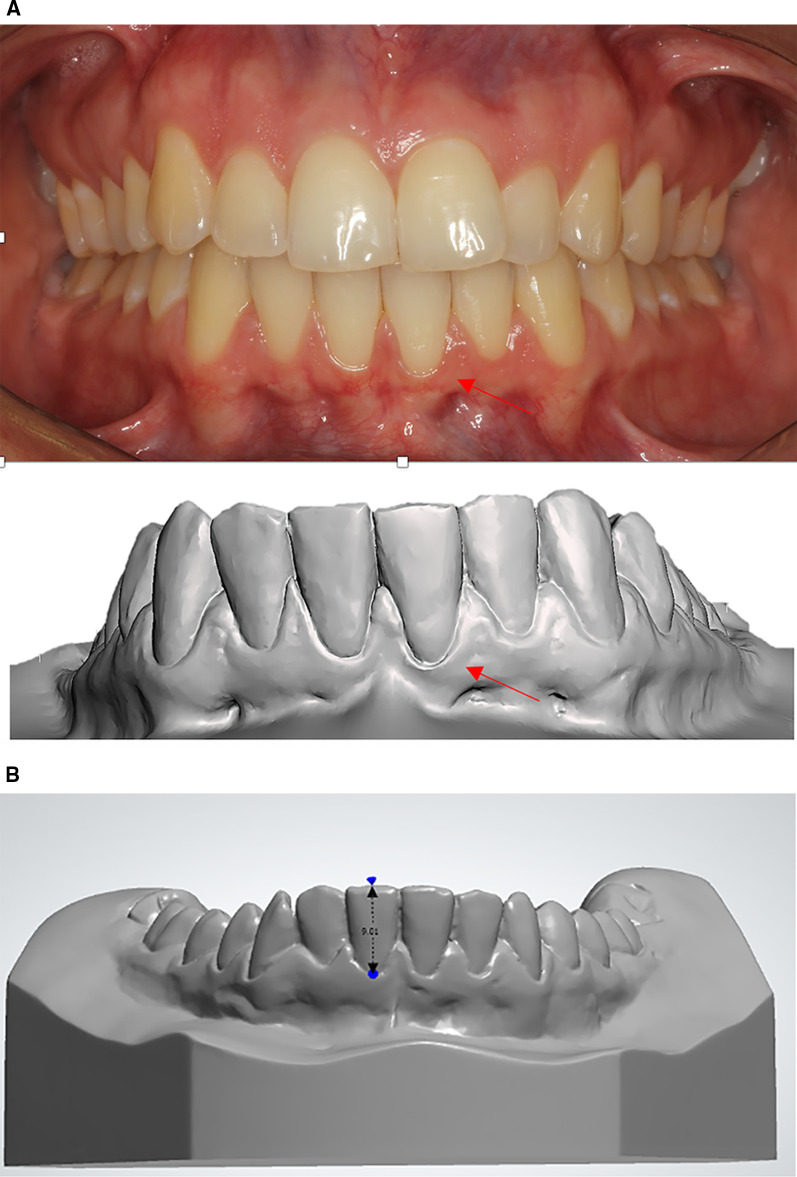
**A** Evaluation of the presence of gingival recession: presence of apical displacement of the gingiva below the cementoenamel junction or the labial margin was clearly below the marginal level of the adjacent teeth. **B** Clinical crown height was measured as a linear measurement between the most apical region of the gingiva and the incisal edge of the mandibular incisor

To evaluate the presence of gingival recession, intraoral photographs and digital dental models were analyzed. The presence of gingival recession was assessed through visual inspection on the labial aspect of the mandibular incisors. Gingival recession was considered present when the gingival margin was located apically to the cementoenamel junction or when the gingival margin was markedly below the level of the adjacent teeth [17, 18] (Fig. 1A).

### Clinical crown height

The dental models of all patients were digitalized using a 3Shape R700 3D scanner (3Shape A/S, Copenhagen, Denmark). Measurements on the digital dental models were performed using Ortho Analyzer 3D software (3Shape A/S, Copenhagen, Denmark) by only one operator (W.M.).

Clinical crown height was measured in the mandibular incisors in all time points. Clinical crown height was defined as the distance between the deepest point of the gingival margin and the incisal edge (Fig. 1B). The height of gingival recession was considered as the interphase changes in clinical crown height [19].

### Error study

For the error study, 30% of the sample was randomly selected and remeasured by the same examiner (W.M.) after a period of 3 weeks. Random errors were calculated using the Dahlberg’s formula, and systematic errors were evaluated using dependent t tests, at *P* < 0.05 [20].

### Statistical analysis

Sample normal distribution was evaluated using Shapiro–Wilk tests. The frequency of gingival recession was calculated. Chi-square test was used to evaluate the sexual dimorphism, and McNemar’s test was used to compare the frequency of gingival recession between the time points. Changes in mandibular incisor position and clinical crown height were evaluated using dependent t tests. Correlation between gingival recession height and position of the mandibular incisors was evaluated using Pearson correlation test. All statistical analyses were performed with SPSS statistical software (version 23.0, IBM Corp., Armonk, N.Y., USA). The level of significance regarded was 5%.

## Results

The random errors ranged between 0.15 mm (clinical crown height) and 0.43 mm (L1 to Apo) for linear measurements and between 1.66° (L1-NB) and 1.8° (IMPA) for angular measurements. No variables showed statistically significant systematic errors.

Mandibular incisor inclination and protrusion significantly increased during treatment (T2–T1) and in the complete follow-up period (T3–T1). Clinical crown height increased in the posttreatment period (T3–T2) and complete follow-up period (T3–T1, Table 2).

**Table 2:** Changes in mandibular incisor inclination and clinical crown height (gingival recession) in the three time point intervals (dependent t tests)

	T1		T2		T3		*T2–T1*			*T3–T2*			*T3–T1*		
	Mean	SD	Mean	SD	Mean	SD	Mean	SD	*P*	Mean	SD	*P*	Mean	SD	*P*
IMPA (L1-MP) (∫)	99.39	7.20	103.48	7.71	104.56	8.04	4.09	4.84	0.011*	1.09	4.52	0.351	5.18	4.92	0.000*
L1-NB (∫)	28.72	5.54	32.77	5.24	31.84	5.78	4.05	4.57	0.003*	− 0.93	3.82	0.345	3.12	5.08	0.018*
L1-NB (mm)	5.31	2.20	6.54	2.54	6.24	2.47	1.23	1.41	0.003*	− 0.91	1.15	0.312	0.93	1.41	0.011*
L1 to A-Po (mm)	1.46	1.59	3.11	2.04	2.42	1.43	1.64	2.50	0.019*	− 0.69	2.07	0.205	0.96	1.54	0.023*
Clinical crown height (mm)	7.32	0.80	7.30	0.99	7.78	0.94	− 0.03	0.65	0.873	0.49	0.91	0.049*	0.46	0.80	0.020*

T1—Pretreatment; T2—Posttreatment; T3—Follow-up

*Statistically significant at *P* < 0.05

The presence of gingival recession was observed in 6 (9.4%), 12 (18.8%) and 24 (37.5%) mandibular incisors at the pretreatment, posttreatment and follow-up stages, respectively (Table 3). No difference was observed between males and females for the frequency of gingival recessions **(**T1, *P* = 0.119, T2, *P* = 0.197 and T3, *P* = 0.513). The frequency of gingival recession increased over time, and the increase was statistically significant from T2 to T3 and from T1 to T3 (Table 4).

**Table 3:** Frequency of gingival recessions before treatment, after treatment and at follow-up (64 teeth)

Recession status	Teeth 32			Teeth 31			Teeth 41			Teeth 42			Mandibular incisors			Patients		
	T1	T2	T3	T1	T2	T3	T1	T2	T3	T1	T2	T3	T1	T2	T3	T1	T2	T3
Recession	0	0	2	3	4	9	3	7	9	0	1	4	6 (9.4%)	12 (18.8%)	24 (37.5%)	4 (25%)	7 (43.8%)	11 (68.8%)
No recession	16	16	14	13	12	7	13	9	7	16	15	12	58 (90.6%)	52 (81.2%)	40 (62.5%)	12 (75%)	9 (56.2%)	5 (31.2%)

T1—Pretreatment; T2—Posttreatment; T3—Follow-up

**Table 4:** Interphase changes in the frequency of mandibular incisor gingival recession (McNemar’s tests)

Recession status	Time								
	*T1*	*T2*	*P*	*T2*	*T3*	*P*	*T1*	*T3*	
Presence of gingival recession	6	12	0.070	12	24	0.008*	6	24	0.000*
Absence of gingival recession	58	52		52	40		58	40	

T1—Pretreatment; T2—Posttreatment; T3—Follow-up

*Statistically significant at *P* < 0.05

No significant correlation between labial inclination or protrusion of the mandibular incisors and clinical crown height was found (Table 5).

**Table 5:** Correlation between mandibular incisors inclination and clinical crown height (gingival recession) in the three time point intervals (Pearson’s correlation analysis)

	Clinical crown height (T2–T1)		Clinical crown height (T3–T2)		Clinical crown height (T3–T1)	
	Coefficient	*P*	Coefficient	*P*	Coefficient	*P*
IMPA (L1-MP) (∫)	0.31	0.239	0.19	0.472	0.12	0.959
L1-NB (∫)	0.21	0.443	0.21	0.442	0.14	0.560
L1-NB (mm)	0.38	0.147	0.29	0.276	0.26	0.261
L1 to A-Po (mm)	0.16	0.553	0.12	0.671	0.16	0.504

T1—Pretreatment; T2—Posttreatment; T3—Follow-up

## Discussion

The null hypothesis was accepted. There was no correlation between mandibular incisor changes after comprehensive orthodontic treatment using the Jasper Jumper appliance and the increase in gingival recession in the long term (Table 5). The possible explanation is that labial tipping and gingival apical migration are not simultaneal occurrences. Our results showed that labial movement of the mandibular incisors occurs during orthodontic treatment, while the increases in clinical crown height are a posttreatment occurrence. To our knowledge, no previous study evaluated the correlation between changes in clinical crown height and position of mandibular incisors in the long term, after using Jasper Jumper therapy. Most studies considered only the period of orthodontic treatment and observed no correlation between the labial movement of the mandibular incisors and gingival recession occurrence [17, 18, 21–23]. On the other hand, some studies observed the aforementioned relation demonstrating the variability in periodontal responses [24, 25]. Previous systematic reviews concluded that contradictory results on the association of labial inclination of mandibular incisors and gingival recessions were found, and future studies are needed to clarify these controversies [25, 26].

To the best of our knowledge, the present research is the first study with a 10-year follow-up observing the development of gingival recessions after comprehensive orthodontic treatment using the Jasper Jumper appliance retained with Class II elastics. The methodology used in this study was based on previous studies [17, 18]. The influence of maturational changes in the clinical crown height over time could have occurred. However, a previous study evaluated the aging changes in subjects with normal occlusion over a 40-year period and observed no significant changes in the clinical crown height of the mandibular central incisors as the apical migration of the gingival margin was similar to the incisal wear [27]. In our study, patients were evaluated after 11.90 years of debonding and patients with bruxism or dental wear facets were not included in the study.

Labial inclination and protrusion of the mandibular incisors increased after Jasper Jumper therapy and remained stable in the follow-up period (Table 2). T2–T1 changes in mandibular incisor are due to both Jasper Jumper therapy and Class II elastics together. These outcomes were expected as a dental effect promoted by most fixed functional appliances, independent of the growth phase [20, 28]. Studies observed similar results when the Jasper Jumper appliance was used followed by comprehensive orthodontic treatment [29–31]. A previous systematic review with the Jasper Jumper appliance and other fixed functional appliances showed even higher labial tipping of the mandibular incisors, probably because patients were evaluated immediately after functional appliance removal. In our study, the patients were evaluated after the comprehensive orthodontic treatment [2].

The frequency of gingival recession increased after treatment during the follow-up period (Tables 3, 4). The increase in clinical crown height also increased from debonding to the 11-year follow-up stage, confirming an apical displacement of the gingival margin (Table 2). Gingival recessions have multifactorial etiological background, and Class II treatment with functional appliance could represent a predisposing factor. Proclination of the mandibular incisors during fixed functional appliance treatment might create areas with bone dehiscences, increasing the risk of gingival recessions in the long term [32–34]. In addition, mandibular incisors are covered by a very thin labial bone plate [35–37]. On the other hand, a study evaluating 225 regular dental care participants at baseline and after 5 years and 12 years of follow-up showed that the percentage of participants with gingival recessions increased over time [38]. Although previous studies have reported weak correlations between gingival recessions and aging, some degree of cumulative effect over time from primary causes and predisposing factors might occur [9, 39]. Another factor that might have influenced the increase in gingival recessions in the long term was the mandibular fixed retainer. However, a previous study by Juloski et al. [40] showed that the long-term presence of fixed lingual retainers did not seem to increase the development of labial gingival recessions in the mandibular incisors. In this study, three patients did not have fixed lingual retainers at T3. Among these three patients, one patient had no changes in clinical crown height (− 0.01 mm), the other patient had a change similar to the average (0.62 mm), and the third one had a change greater than the average (0.98 mm) in clinical crown height from T1 to T3.

Previous studies with the Herbst appliance are in accordance with our results showing no increase in gingival recession during the therapy period [13, 18, 41]. Only one study evaluated the frequency of gingival recessions long term after the Herbst appliance [13]. This longitudinal study evaluated patients after 32 years of debonding and demonstrated minor gingival recessions in a few subjects [13].

When gingival recession is observed in the posttreatment period of comprehensive orthodontic treatment using the Jasper Jumper appliance, factors like the presence of chronic trauma, chronic inflammatory periodontal disease and occlusal trauma (occlusal interference) should be investigated and an interdisciplinary treatment is needed [9]. Future longitudinal studies should be performed to compare the gingival recessions in patients treated with Jasper Jumper therapy and a comparison group treated with fixed appliances only.

The main limitation of this study was the lack of a control group of nontreated Class II malocclusion patients. Some changes in the follow-up period might have occurred due to age-related changes, and results should be considered with caution. Additionally, evaluation of the correlation between changes in mandibular incisor position and in clinical crown height was the main goal of this study.

## Conclusion

- Mandibular incisor inclination and protrusion significantly increased during treatment and maintained stable during the follow-up.
- Both the frequency of gingival recession and the clinical crown height increased only during the posttreatment follow-up period of comprehensive orthodontic treatment using the Jasper Jumper therapy.
- No significant correlation between the amount of labial movement of the mandibular incisor and clinical crown height increase was found.

## Data Availability

The data underlying this article are available in the article and available from the corresponding author on reasonable request.

## References

[1] Cançado RH, Pinzan A, Janson G, Henriques JFC, Neves LS, Canuto CE (2008). Occlusal outcomes and efficiency of 1-and 2-phase protocols in the treatment of Class II Division 1 malocclusion. Am J Orthod Dentofacial Orthop.

[2] Zymperdikas VF, Koretsi V, Papageorgiou SN, Papadopoulos MA (2015). Treatment effects of fixed functional appliances in patients with Class II malocclusion: a systematic review and meta-analysis. Eur J Orthod.

[3] Ishaq RAR, AlHammadi MS, Fayed MM, El-Ezz AA, Mostafa Y (2016). Fixed functional appliances with multibracket appliances have no skeletal effect on the mandible: A systematic review and meta-analysis. Am J Orthod Dentofac Orthop.

[4] Perinetti G, Primozic J, Furlani G, Franchi L, Contardo L (2015). Treatment effects of fixed functional appliances alone or in combination with multibracket appliances: a systematic review and meta-analysis. Angle Orthod.

[5] Aras I, Pasaoglu A (2017). Class II subdivision treatment with the Forsus Fatigue Resistant Device vs intermaxillary elastics. Angle Orthod.

[6] Janson G, Sathler R, Fernandes TMF, Branco NCC, de Freitas MR (2013). Correction of Class II malocclusion with Class II elastics: a systematic review. Am J Orthod Dentofac Orthop.

[7] Jones G, Buschang PH, Kim KB, Oliver DR (2008). Class II non-extraction patients treated with the Forsus Fatigue Resistant Device versus intermaxillary elastics. Angle Orthod.

[8] Nelson B, Hägg U, Hansen K, Bendeus M (2007). A long-term follow-up study of Class II malocclusion correction after treatment with Class II elastics or fixed functional appliances. Am J Orthod Dentofac Orthop.

[9] Jati AS, Furquim LZ, Consolaro A (2016). Gingival recession: its causes and types, and the importance of orthodontic treatment. Dent Press J Orthod.

[10] Sharma K, Mangat S, Kichorchandra M, Handa A, Bindhumadhav S, Meena M (2017). Correlation of orthodontic treatment by fixed or myofunctional appliances and periodontitis: a retrospective study. J Contemp Dent Pract.

[11] Boke F, Gazioglu C, Akkaya S, Akkaya M (2014). Relationship between orthodontic treatment and gingival health: a retrospective study. Eur J Dent.

[12] Garib DG, Yatabe MS, Ozawa TO, Silva Filho OGD (2010). Alveolar bone morphology under the perspective of the computed tomography: defining the biological limits of tooth movement. Dent Press J Orthod.

[13] Pancherz H, Bjerklin K (2014). Mandibular incisor inclination, tooth irregularity, and gingival recessions after Herbst therapy: a 32-year follow-up study. Am J Orthod Dentofac Orthop.

[14] Moinester M, Gottfried R (2014). Sample size estimation for correlations with pre-specified confidence interval. Quant Methods Psychol.

[15] Baccetti T, Franchi L, McNamara JA (2005). The cervical vertebral maturation (CVM) method for the assessment of optimal treatment timing in dentofacial orthopedics. Semin Orthod.

[16] Foncatti CF, Castanha Henriques JF, Janson G, Caldas W, Garib DG (2017). Long-term stability of Class II treatment with the Jasper jumper appliance. Am J Orthod Dentofac Orthop.

[17] Allais D, Melsen B (2003). Does labial movement of lower incisors influence the level of the gingival margin? A case–control study of adult orthodontic patients. Eur J Orthod.

[18] Ruf S, Hansen K, Pancherz H (1998). Does orthodontic proclination of lower incisors in children and adolescents cause gingival recession?. Am J Orthod Dentofac Orthop.

[19] Villard NM, Patcas R (2015). Does the decision to extract influence the development of gingival recessions? A retrospective long-term evaluation. J Orofac Orthop.

[20] de Lima KJRS, Henriques JFC, Janson G, da Costa Pereira SC, Neves LS, Cançado RH (2013). Dentoskeletal changes induced by the Jasper jumper and the activator-headgear combination appliances followed by fixed orthodontic treatment. Am J Orthod Dentofac Orthop.

[21] Djeu G, Hayes C, Zawaideh S (2002). Correlation between mandibular central incisor proclination and gingival recession during fixed appliance therapy. Angle Orthod.

[22] Melsen B, Allais D (2005). Factors of importance for the development of dehiscences during labial movement of mandibular incisors: a retrospective study of adult orthodontic patients. Am J Orthod Dentofacial Orthop.

[23] Renkema A-M, Navratilova Z, Mazurova K, Katsaros C, Fudalej PS (2014). Gingival labial recessions and the post-treatment proclination of mandibular incisors. Eur J Orthod.

[24] Yared KF, Zenobio EG, Pacheco W (2006). Periodontal status of mandibular central incisors after orthodontic proclination in adults. Am J Orthod Dentofac Orthop.

[25] Ciavarella D, Tepedino M, Gallo C, Montaruli G, Zhurakivska K, Coppola L (2017). Post-orthodontic position of lower incisors and gingival recession: a retrospective study. J Clin Exp Dent.

[26] Aziz T, Flores-Mir C (2011). A systematic review of the association between appliance-induced labial movement of mandibular incisors and gingival recession. Aust Orthod J.

[27] Massaro C, Miranda F, Janson G, de Almeida RR, Pinzan A, Martins DR (2018). Maturational changes of the normal occlusion: a 40-year follow-up. Am J Orthod Dentofac Orthop.

[28] Koretsi V, Zymperdikas VF, Papageorgiou SN, Papadopoulos MA (2014). Treatment effects of removable functional appliances in patients with Class II malocclusion: a systematic review and meta-analysis. Eur J Orthod.

[29] Bassarelli T, Franchi L, Defraia E, Melsen B (2016). Dentoskeletal effects produced by a Jasper Jumper with an anterior bite plane. Angle Orthod.

[30] Herrera FS, Henriques JFC, Janson G, Francisconi MF, de Freitas KMS (2011). Cephalometric evaluation in different phases of Jasper jumper therapy. Am J Orthod Dentofac Orthop.

[31] Kucukkeles N, Ilhan I, Orgun IA (2007). Treatment efficiency in skeletal Class II patients treated with the jasper jumper. Angle Orthod.

[32] Schwartz JP, Raveli TB, Schwartz-Filho HO, Raveli DB (2016). Changes in alveolar bone support induced by the Herbst appliance: a tomographic evaluation. Dent Press J Orthod.

[33] Garlock DT, Buschang PH, Araujo EA, Behrents RG, Kim KB (2016). Evaluation of marginal alveolar bone in the anterior mandible with pretreatment and posttreatment computed tomography in nonextraction patients. Am J Orthod Dentofac Orthop.

[34] Yu Q, Pan XG, Ji GP, Shen G (2009). The association between lower incisal inclination and morphology of the supporting alveolar bone–a cone-beam CT study. Int J Oral Sci.

[35] Fuhrmann R (1996). Three-dimensional interpretation of labiolingual bone width of the lower incisors. Part II. J Orofac Orthop.

[36] Baysal A, Ucar FI, Buyuk SK, Ozer T, Uysal T (2013). Alveolar bone thickness and lower incisor position in skeletal Class I and Class II malocclusions assessed with cone-beam computed tomography. Korean J Orthod.

[37] Nauert K, Berg R (1999). Evaluation of labio-lingual bony support of lower incisors in orthodontically untreated adults with the help of computed tomography. J Orofac Orthop.

[38] Serino G, Wennstrom JL, Lindhe J, Eneroth L (1994). The prevalence and distribution of gingival recession in subjects with a high standard of oral hygiene. J Clin Periodontol.

[39] Mckenna G, Burke FM (2010). Age-related oral changes. Dent Update.

[40] Juloski J, Glisic B, Vandevska-Radunovic V (2017). Long-term influence of fixed lingual retainers on the development of gingival recession: a retrospective, longitudinal cohort study. Angle Orthod.

[41] Bock NC, Ruf S, Wiechmann D, Jilek T (2016). Herbst plus Lingual versus Herbst plus Labial: a comparison of occlusal outcome and gingival health. Eur J Orthod.

